# Natural products as a source of Coronavirus entry inhibitors

**DOI:** 10.3389/fcimb.2024.1353971

**Published:** 2024-02-21

**Authors:** Dávid Szabó, Andrew Crowe, Cyril Mamotte, Padraig Strappe

**Affiliations:** ^1^ Curtin Health Innovation Research Institute, Curtin University, Bentley, WA, Australia; ^2^ Curtin Medical School, Curtin University, Bentley, WA, Australia

**Keywords:** coronavirus, SARS-CoV-2, COVID-19, antiviral, entry inhibitor, fusion inhibitor, natural product

## Abstract

The COVID-19 pandemic has had a significant and lasting impact on the world. Four years on, despite the existence of effective vaccines, the continuous emergence of new SARS-CoV-2 variants remains a challenge for long-term immunity. Additionally, there remain few purpose-built antivirals to protect individuals at risk of severe disease in the event of future coronavirus outbreaks. A promising mechanism of action for novel coronavirus antivirals is the inhibition of viral entry. To facilitate entry, the coronavirus spike glycoprotein interacts with angiotensin converting enzyme 2 (ACE2) on respiratory epithelial cells. Blocking this interaction and consequently viral replication may be an effective strategy for treating infection, however further research is needed to better characterize candidate molecules with antiviral activity before progressing to animal studies and clinical trials. In general, antiviral drugs are developed from purely synthetic compounds or synthetic derivatives of natural products such as plant secondary metabolites. While the former is often favored due to the higher specificity afforded by rational drug design, natural products offer several unique advantages that make them worthy of further study including diverse bioactivity and the ability to work synergistically with other drugs. Accordingly, there has recently been a renewed interest in natural product-derived antivirals in the wake of the COVID-19 pandemic. This review provides a summary of recent research into coronavirus entry inhibitors, with a focus on natural compounds derived from plants, honey, and marine sponges.

## 1 Introduction

COVID-19 is a highly transmissible viral infection that spreads via respiratory droplets (World Health Organization, n.d.a). Originating in Wuhan, China as a localized outbreak of pneumonia, the disease rapidly became a global threat, leading the World Health Organization (WHO) to declare a Public Health Emergency of International Concern (PHEIC) in January 2020 (Lu et al., 2020; World Health Organization, 2020; Wu et al., 2020; Zhou et al., 2020). While most cases of COVID-19 present as mild respiratory illness, some can progress to severe pneumonia and multi-organ injury, particularly in high-risk individuals such as older people and those with underlying health conditions (Gupta et al., 2020; World Health Organization, n.d.a; Xu et al., 2020). Furthermore, some individuals may develop a range of long-term symptoms including fatigue and sensory confusion which contribute to the syndrome known as ‘Long Covid’ (Centers for Disease Control and Prevention, n.d.a). The average global mortality of COVID-19 is approximately 2%, which is significantly lower than that of the 2003 SARS pandemic (11%) and the outbreak of Middle East respiratory syndrome (MERS) in 2012 (35%) (Chan-Yeung and Xu, 2003; Johns Hopkins University & Medicine, n.d.; World Health Organization, n.d.b; World Health Organization, n.d.c). Despite this, SARS-CoV-2 has higher overall transmissibility due to significant airborne exposure (Sills et al., 2020). As of November 2023, there have been 771 million confirmed cases of COVID-19 across the globe and 6.9 million associated deaths (World Health Organization, n.d.d). Recently, the PHEIC status of COVID-19 was downgraded in response to decreasing trends in hospitalizations and deaths, and the WHO shifted their focus to the long-term management of the disease, listing ongoing prevention, diagnosis, and treatment efforts amongst their main strategic objectives (World Health Organization, 2023a; World Health Organization, 2023b). These efforts remain vital to protecting susceptible individuals around the world, including the estimated 70% of people in low-income countries who remain unvaccinated against COVID-19 (Mathieu et al., 2020). As such, there is an ongoing need for further research into the development of antiviral therapies for coronavirus infection.

### 1.1 Severe acute respiratory syndrome coronavirus 2 (SARS-CoV-2)

**Figure 1:**
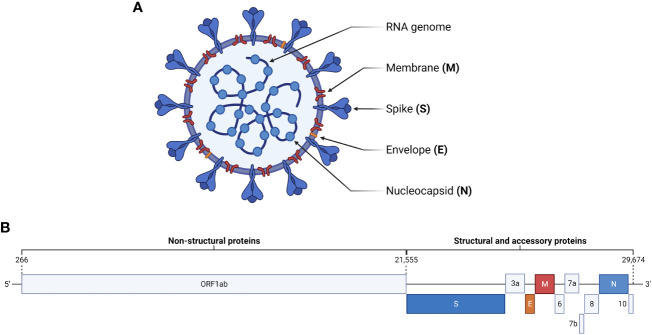
SARS-CoV-2 virion and genome structure. **(A)** The SARS-CoV-2 virion consists of a positive-sense, single stranded RNA genome packaged by nucleocapsid proteins. Embedded in the surrounding viral membrane are membrane and envelope proteins, which contribute to pathogenicity, and spike proteins, which facilitate viral entry by interacting with cellular ACE2. **(B)** The genome of SARS-CoV-2 is ~30 kb and consists of 12 open reading frames. ORF1ab encodes non-structural proteins, while the remaining ORFs encode the major structural proteins and accessory proteins involved in pathogenesis.

The causative agent of COVID-19 is SARS-CoV-2, a member of the *Sarbecovirus* subgenus of betacoronaviruses (**Figure 1A**) (Chan et al., 2020; Gorbalenya et al., 2020; Wu et al., 2020). Other betacoronaviruses include SARS-CoV, MERS-CoV, and some of the viruses responsible for the common cold such as HCoV-OC43 and HCoV-229E (Lefkowitz et al., 2017). The ~30 kb positive sense, single-stranded RNA genome of SARS-CoV-2 consists of 12 open reading frames (ORFs) (**Figure 1B**) (Wu et al., 2020). The replicase ORF (ORF1ab) makes up the first two-thirds of the genome and encodes 16 non-structural proteins (NSPs) involved in viral replication (Chan et al., 2020; Fehr and Perlman, 2015). The remaining ORFs encode accessory proteins involved in pathogenesis and the spike (S), envelope (E), membrane (M), and nucleocapsid (N) structural proteins common to all coronaviruses (Fehr and Perlman, 2015; Redondo et al., 2021; V’kovski et al., 2021). The genome is bounded by a 5’ cap and 3’ poly-A tail, allowing for direct translation by host ribosomes (Fehr and Perlman, 2015).

Coronaviruses are zoonotic and are endemic in other species including cats, ferrets, and bats (Li et al., 2005; Hu et al., 2018; Shi et al., 2020). Given that SARS-CoV-2 shares 96.2% sequence identity with the Chinese bat coronavirus RaTG13, it is proposed that bats were the original reservoir for COVID-19 (Zhou et al., 2020; World Health Organization, 2021a; Lytras et al., 2022). Furthermore, a comprehensive study by the WHO in 2021 concluded that the most likely mode of transmission to humans was via an intermediate host, which current data suggests may have been pangolins (World Health Organization, 2021a; Liu et al., 2020). Like other RNA viruses, SARS-CoV-2 is subject to a high rate of mutation due to the lack of proofreading functionality in RNA-dependent RNA polymerase (RdRp) (Fleischmann, 1996). This rapid evolution drove the emergence of many viral variants, which are classified using the Pango nomenclature system and labelled by the WHO using the Greek alphabet (**Table 1**) (Rambaut et al., 2020; Centers for Disease Control and Prevention, n.d.b; World Health Organization, 2021b). Notable mutations facilitating the emergence of new variants include N501Y and D614G, which contributed to higher transmissibility, and E484K, which helped the virus avoid neutralization by the immune system (Jangra et al., 2021; Plante et al., 2021; Zhou et al., 2021; Liu et al., 2022). Additionally, new variants can emerge by genetic recombination between two lineages infecting the same cell at the same time (Focosi and Maggi, 2022). As of December 2023, the major circulating variants of interest (VOI) are the XBB.1.5, XBB.1.16, EG.5 (‘Eris’), B.A.2.86 (‘Pirola’), and JN.1 recombinant subvariants of Omicron (World Health Organization, n.d.e; Dyer, 2023; Mahase, 2023).

**Table 1:** List of major SARS-CoV-2 variants (Centers for Disease Control and Prevention, n.d.b; O’Toole et al., 2021; European Centre for Disease Prevention and Control, 2024).

WHO label	Pango lineage	Date first detected	Country first detected	Current status*
Alpha	B.1.1.7	Sep 2020	United Kingdom	VBM
Beta	B.1.351	Sep 2020	South Africa	VBM
Gamma	P.1	Dec 2020	Brazil	VBM
Delta	B.1.617.2	Dec 2020	India	VBM
Epsilon	B.1.427/B.1.429	Sep 2020	USA	VBM
Zeta	P.2	Jan 2021	Brazil	VBM
Eta	B.1.525	Dec 2020	Nigeria	VBM
Theta	P.3	Jan 2021	The Philippines	VBM
Iota	B.1.526	Dec 2020	USA	VBM
Kappa	B.1.617.1	Dec 2020	India	VBM
Lambda	C.37	Dec 2020	Peru	VBM
Mu	B.1.621	Jan 2021	Colombia	VBM
Omicron	B.1.1.529	N/A	N/A	VBM
Omicron	BA.1	Nov 2021	South Africa, Botswana	VBM
Omicron	BA.2.86	N/A	N/A	VOI
Omicron	XBB	N/A	N/A	VBM
Omicron	XBB.1.5	N/A	N/A	VOI
Omicron	XBB.1.16	N/A	N/A	VBM

*VOC, Variant of Concern; VOI, Variant of Interest; VBM, Variant Being Monitored. N/A, not available.

### 1.2 SARS-CoV-2 life cycle

**Figure 2:**
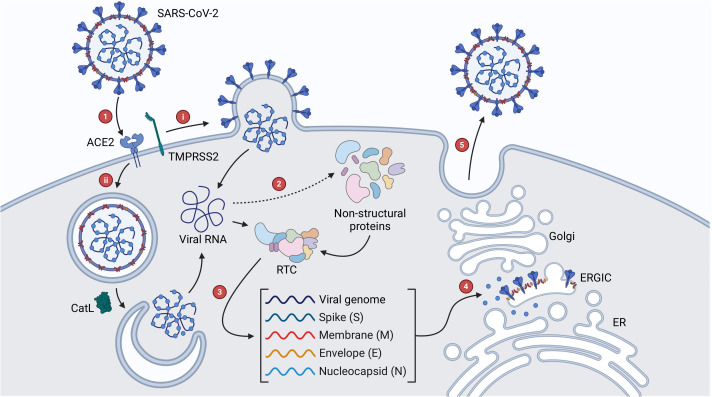
SARS-CoV-2 viral life cycle. (1) Interaction of the S protein with ACE2 initiates viral entry, which proceeds via **(A)** TMPRSS2-mediated fusion of the viral and cellular membranes or **(B)** endocytosis and CatL-mediated fusion of the viral and endosomal membranes. (2) Once the genome enters the cytoplasm, ORF1ab is expressed as the polyproteins pp1a and pp1ab which are cleaved by viral proteases into NSPs that make up the RTC. (3) The remainder of the genome is transcribed by the RTC into a nested set of subgenomic RNAs, which are translated to form structural and accessory proteins. (4) Proteins are translocated to the ERGIC where assembly begins. (5) Viral progeny are translocated to the cell membrane and released by exocytosis.

The first stage in the life cycle of SARS-CoV-2 is fusion and entry, which begins with the interaction of the S protein and angiotensin converting enzyme 2 (ACE2) in the upper respiratory tract (**Figure 2**) (Zhou et al., 2020; V’kovski et al., 2021). ACE2 is expressed in a decreasing gradient from the upper to lower respiratory tract, a pattern which reflects the progression of SARS-CoV-2 infection from the nasopharynx to the lungs (Hou et al., 2020). As part of the renin-angiotensin system, ACE2 helps maintain blood pressure by stimulating vasodilation in response to the vasoconstriction effected by angiotensin converting enzyme (ACE) (Salamanna et al., 2020). As such, ACE2 is expressed in multiple organs, which contributes to the multi-organ injury experienced in severe cases of COVID-19 (Ni et al., 2020). The exact method of SARS-CoV-2 entry depends on the level of transmembrane serine protease 2 (TMPRSS2) expression on the host cell. If there is sufficient TMPRSS2, this enzyme cleaves the S protein at a specific site termed S2′ and exposes a fusion peptide responsible for facilitating genome release into the cytoplasm through fusion with the cell membrane (Hoffmann et al., 2020b; Jackson et al., 2022). In contrast, if there is insufficient TMPRSS2, the virus is endocytosed and the S2′ site is cleaved by cathepsin L (CatL) following endosomal acidification, enabling fusion between the viral and endosomal membranes (Bosch et al., 2008; Bayati et al., 2021; Jackson et al., 2022). Once in the cytoplasm, the viral nucleocapsid dissociates from the RNA genome and a ribosomal frameshift (-1) allows translation of ORF1ab into the polyproteins pp1a and pp1ab (Fehr and Perlman, 2015; V’kovski et al., 2021). Viral proteases papain-like protease (PLpro) and 3-chymotrypsin like protease (3CLpro) are then expressed and post-translationally cleave these polyproteins into 16 NSPs that make up the viral replication-transcription complex (RTC) (Fehr and Perlman, 2015; V’kovski et al., 2021). This complex transcribes the remainder of the viral genome into a nested set of subgenomic mRNAs which are translated to form structural and accessory proteins. These proteins are then translocated to the endoplasmic reticulum-Golgi intermediate compartment (ERGIC) where assembly occurs (V’kovski et al., 2021). The oligomerization of M proteins in the ERGIC membrane initiates curvature and is the first step in the assembly of viral progeny (Fehr and Perlman, 2015; Zhang et al., 2022). In a competent virus, M proteins are also involved in the suppression of interferon production, which weakens the immune response and encourages viral replication (Zheng et al., 2020). Similarly, E proteins assist in assembly and function as ion channels that contribute to pathogenicity through the stimulation of acute respiratory stress (Fehr and Perlman, 2015; Mandala et al., 2020; Xia et al., 2021). N proteins package the viral genome, forming a ribonucleoprotein complex, which is then recruited by M proteins to the membrane during assembly (Fehr and Perlman, 2015; Zeng et al., 2020; Zhang et al., 2022). S proteins are translocated to the ERGIC where they integrate into the membrane and are cleaved by furin protease into S1 and S2 subunits, which in a competent virus are responsible for ACE2 binding and membrane fusion respectively (Fehr and Perlman, 2015; Hoffmann et al., 2020a; V’kovski et al., 2021). Finally, viral progeny bud off from the ERGIC membrane and are released from the infected cell by exocytosis (Fehr and Perlman, 2015).

### 1.3 Current antivirals for COVID-19

Compared to vaccines, which are administered prophylactically, antivirals are used to reduce disease severity post-infection. While advances in vaccine technology enabled the rapid production and approval of COVID-19 vaccines, the availability of antivirals has so far been largely reliant on drug repurposing due to the long timeframe required for *de novo* development. One of the first drugs repurposed for the treatment of COVID-19 was remdesivir, a broad-spectrum antiviral which functions by incorporating into the RNA strand being newly synthesized by viral RdRp in place of adenosine, stalling the enzyme and disrupting RNA replication (U.S. Food & Drug Administration, 2020a; Kokic et al., 2021). This drug effectively attenuated wild-type SARS-CoV-2 infection *in vitro* (EC_50 = _0.77 µM), and appeared to function post virus entry, supporting its purported function as a nucleoside analogue (Wang et al., 2020). Furthermore, clinical trials suggest that remdesivir improves clinical outcomes for people with COVID-19, though the extent of this improvement appears to be highly variable between studies. The results of one trial indicated a five-day reduction in recovery time in patients receiving remdesivir compared to patients receiving a placebo, while another reported that individuals receiving a 3-day course of remdesivir had an 87% lower risk of hospitalization or death compared to a control group (Beigel et al., 2020; Gottlieb et al., 2021). In contrast, the WHO Solidarity trial investigating the efficacy of repurposed drugs against COVID-19 concluded that remdesivir only marginally reduced the risks of mortality and requiring mechanical ventilation in patients with COVID-19, and had no significant effect on patients already receiving ventilation (Solidarity Trial Consortium. Remdesivir, 2022). Other repurposed antivirals include molnupiravir (Lagevrio), which causes an accumulation of errors throughout the viral genome following incorporation into replicating viral RNA as a cytidine analogue, and nirmatrelvir-ritonavir (Paxlovid), which inhibits the SARS-CoV-2 protease 3CLpro (Saravolatz et al., 2022). While molnupiravir has been shown to shorten the duration of viraemia in some patients, clinical trials have reported opposing results regarding its efficacy in reducing the risks of hospitalization and death (Jayk Bernal et al., 2021; Fischer et al., 2022; Butler et al., 2023). Furthermore, while multiple trials have reported the efficacy of nirmatrelvir-ritonavir in reducing these risks, a recent study reported no significant reduction in mortality risk in adult patients with comorbidities such as cardiovascular disease and chronic liver disease (Bajema et al., 2023; Dryden-Peterson et al., 2023; Liu et al., 2023; Schwartz et al., 2023).

Furthermore, the attempted repurposing of other drugs for use as COVID-19 antivirals resulted in widespread inefficacy and adverse effects. For example, the antimalarial drug chloroquine was previously shown to inhibit SARS-CoV infection by increasing endosomal pH and interfering with the glycosylation of ACE2, and *in vitro* studies reported a similar antiviral effect on wild-type SARS-CoV-2 (EC_50 = _1.13 µM) (Vincent et al., 2005; Wang et al., 2020). However, multiple clinical trials reported no benefit of chloroquine or its derivative hydroxychloroquine for the treatment of COVID-19, and the results of one study indicated that people hospitalized with COVID-19 that received hydroxychloroquine had a higher likelihood of death or requiring mechanical ventilation compared to those receiving no treatment (Horby et al., 2020; Axfors et al., 2021). As a result, the emergency use authorizations for chloroquine and hydroxychloroquine were withdrawn by the FDA shortly after they were granted (U.S. Food & Drug Administration, 2020b). Similarly, the antiparasitic drug ivermectin showed strong *in vitro* efficacy and reduced the amount of wild-type SARS-CoV-2 viral RNA in infected Vero cells ~5000-fold 48 hours after treatment (IC_50_ = ~2 µM) (Caly et al., 2020). However, multiple clinical trials reported no influence of ivermectin on the risk of disease progression or hospitalization (Lim et al., 2022; Reis et al., 2022). Thus, while COVID-19 vaccines are now widely available, there are currently few effective purpose-built antivirals to protect individuals at risk of severe disease. Furthermore, recent studies suggest that some people treated with currently available antivirals remain susceptible to a resurgence of COVID-19 symptoms and infection, a phenomenon termed viral rebound (Anderson et al., 2022; Boucau et al., 2022; Charness et al., 2022; Coulson et al., 2022). Therefore, there remains a need for further research into the development of coronavirus antivirals.

### 1.4 Potential targets for novel coronavirus antivirals

There are multiple potential modes of action for novel coronavirus antivirals. Certain therapies may target aspects of the host cell involved in disease progression including cell surface proteins that facilitate viral entry. For example, the protease inhibitor camostat mesylate was shown to prevent SARS-CoV-2 infection by inhibiting TMRPSS2, the cellular protease that mediates maturation of the spike protein and membrane fusion (Hoffmann et al., 2021). Alternatively, some therapies may attenuate the host response to viral infection, which in diseases such as COVID-19 can be more damaging than the infection itself. For example, Zhu et al. demonstrated that patients with severe COVID-19 receiving treatment in the form of umbilical cord-derived mesenchymal stem cell infusion displayed improved clinical symptoms attributed to beneficial cellular differentiation (Zhu et al., 2021). Changes observed included reduced levels of pro-inflammatory cytokines, promotion of lung tissue repair, modulation of immune cell composition, and sustained production of SARS-CoV-2 antibodies (Zhu et al., 2021). Furthermore, novel antiviral therapies may target structural or functional components of the virus itself. One study characterized the SARS-unique domain of the SARS-CoV-2 protease PLpro and demonstrated its potential as a druggable site using the natural compound theaflavin-3,3′-digallate (Qin et al., 2023). Another study investigated the antiviral activity of porphyrin, a natural bioactive compound, against the SARS-CoV-2 N protein. Using *in silico* analyses, porphyrin-derived carbon nanoparticles were found to inhibit nucleocapsid dimerization, an essential step in the formation of a functional N protein (Fibriani et al., 2023). Furthermore, these nanoparticles exhibited *in vitro* antiviral activity, significantly reducing observable cytopathic effect in infected Vero E6 cells (Fibriani et al., 2023).

Another particularly promising mode of antiviral action is the inhibition of viral entry by blocking the interaction of the S protein with ACE2. Similar drugs used in the treatment of other viral infections are referred to as entry inhibitors or fusion inhibitors and follow the general principle of blocking the interaction of a surface glycoprotein with its respective cellular receptor. For example, the HIV entry inhibitor maraviroc blocks the interaction of the HIV-1 surface glycoprotein gp41 with C-C chemokine receptor type 5 on the host cell, and the fusion inhibitor enfuvirtide prevents gp41 from undergoing structural changes required for membrane fusion (Haqqani and Tilton, 2013). Similarly, umifenovir (Arbidol) has reported activity against influenza viruses by preventing the haemagglutinin surface protein from undergoing conformational changes required for fusion (Kadam and Wilson, 2017). This antiviral technology may be translated to combat coronavirus infection in the form of small molecule drugs capable of inhibiting membrane fusion by disrupting the S-ACE2 interaction, hereafter referred to as S-ACE2 inhibitors. For example, Goc et al. used an antibody-based S-ACE2 inhibitor screening kit to screen a variety of fatty acids and lipid-soluble vitamins for their ability to block the S-ACE2 interaction (Goc et al., 2021a). Such kits are based on the principles of an enzyme-linked immunosorbent assay (ELISA) and involve the addition of a protein-tagged SARS-CoV-2 S protein receptor binding domain (RBD) to ACE2, which is immobilized on a plate in the presence of test compounds or patient sera. Following incubation, the unbound RBD is washed away before the appropriate substrate, antibody, or protein is added to quantify the ACE2-bound RBD remaining in the wells (Tan et al., 2020). Using one such assay, the researchers found that polyunsaturated fatty acids such as eicosatetraenoic acid (EPA), docosahexaenoic acid, linoleic acid, and linolenic acid had potent S-ACE2 inhibitory activity (Goc et al., 2021a). Further analysis revealed that linoleic acid and EPA were capable of inhibiting membrane fusion and the cellular entry of SARS-CoV-2 pseudoviruses. Furthermore, these compounds reduced the activity of TMPRSS2 and CatL, suggesting multifaceted antiviral activity (Goc et al., 2021a). In another study, Lee et al. developed an assay based on the NanoBiT protein-protein interaction system to identify candidates for repurposing as coronavirus S-ACE2 inhibitors from an FDA-approved drug library (Lee et al., 2022). Using this assay, which involved simulation of the S-ACE2 interaction using a cell line expressing ACE2 and recombinant S RBD, the researchers identified nine drugs with inhibitory activity, and through further *in vitro* analyses determined that the HIV-1 reverse transcriptase inhibitor Etravirine and integrase inhibitor Dolutegravir blocked the S-ACE2 interaction of multiple SARS-CoV-2 variants including Omicron (Lee et al., 2022). As the EC_50_ values were significantly lower in subsequent pseudovirus neutralization assays compared to the RBD attachment assays, it is likely that these drugs had other antiviral effects in addition to entry inhibition such as protease inhibition that contribute to their overall efficacy, a hypothesis supported by previous literature (Indu et al., 2020; Lee et al., 2022). Thus, entry inhibition by blocking the S-ACE2 interaction is a unique mechanism of action for novel coronavirus antivirals that may also involve beneficial off-target effects. Additionally, antiviral drugs following this mode of action would not be reliant on intracellular delivery, expanding the range of potential candidate molecules to accommodate greater hydrophilicity. While somewhat similar in concept to neutralizing antibodies (nAbs), which bind to epitopes on the S protein to prevent interaction with ACE2, it is purported that small molecule S-ACE2 inhibitors may be less susceptible to the effects of antigenic variation which can reduce the efficacy of nAbs against newer SARS-CoV-2 variants such as Omicron BA.1 and BA.2 (Zhang et al., 2021; Wilhelm et al., 2022; Planas et al., 2022; Meng et al., 2023). However, extensive research into the efficacy of S-ACE2 inhibitors against variant S proteins is required to assess this hypothesis.

## 2 Drug discovery from natural products

Natural products (NPs) derived from plants, animals, and microbes have been used for thousands of years to treat a range of diseases (Yuan et al., 2016). In fact, traditional medicines continue to be used alongside Western therapeutics in many countries including China, Japan, Korea, and India where they play an integral role in the treatment of various illnesses including respiratory infections (Kim and Song, 2012; Yu et al., 2014; Yuan et al., 2016). Furthermore, NPs have contributed substantially to pharmaceutical development over the past forty years (Newman and Cragg, 2020). For example, the anticancer drug paclitaxel was developed from a compound extracted from the bark of the Pacific yew tree *Taxus brevifolia*, and the chemical precursor to the antiparasitic drug ivermectin, avermectin, was isolated from the soil bacteria *Streptomyces avermitilis (* Wani et al., 1971, Burg et al., 1979). Comparatively, the contribution of NPs to the development of antivirals is less prominent. Most antivirals are either synthetic drugs or antibody therapies, and while NPs have contributed to ~6% of all new antivirals produced since 1981, all of these have some level of synthetic modification (Newman and Cragg, 2020). One such product is shikimic acid, which is the precursor compound to the synthetic influenza antiviral oseltamivir and is naturally found in star anise *(Illicium verum) *(Candeias et al., 2018). This indirect and relatively minor contribution is largely due to constraints on working with NPs complicating the discovery of new bioactive molecules. Firstly, crude NP extracts contain mixtures of organic compounds with varying biochemical properties, which can complicate the identification of lead molecules and the analysis of their bioactivity. Moreover, because certain types of NPs such as honey have only trace amounts of the desired molecules, obtaining them in sufficient quantities for use in pharmaceutical development can be challenging (Cianciosi et al., 2018; Wright, 2019). Additionally, due to the vast number of compounds from NPs reported in the literature, novel discovery often requires the use additional tools termed dereplication techniques to avoid the rediscovery of known compounds (Gaudêncio and Pereira, 2015; Wright, 2019). Despite their limitations, NPs offer several unique advantages that warrant further investigation. The extensive use of certain classes of NP in traditional medicines highlight their safety and efficacy in the treatment of infectious disease (Atanasov et al., 2021). Furthermore, many NPs have clinically advantageous bioactivity as they are shaped by natural selection for optimal interaction with biological systems (Atanasov et al., 2021). These complex interactions may have beneficial off-target effects, potentially revealing alternate antiviral mechanisms. Additionally, some NPs exert additive or synergistic effects when combined with other therapies for a greater overall clinical benefit (Atanasov et al., 2021; Cao et al., 2022). For example, one study screened an extensive library of botanical drugs using a pseudovirus model and found four compounds capable of inhibiting viral entry by blocking membrane fusion. Oleanonic acid, angeloylgomisin O, schisandrin B, and procyanidin all inhibited SARS-CoV-2 infection *in vitro* with respective IC_50_ values of 1.4 µM, 3.7 µM, 7.3 µM, and 33 µM (Cao et al., 2022). Additionally, angeloylgomisin O displayed strong synergistic reduction of viral replication when combined with the RdRp inhibitor remdesivir (Cao et al., 2022). Thus, while historically overlooked in relation to antiviral therapy, NPs contain a wealth of bioactive molecules that may assist in the development of novel antivirals.

**Figure 3:**
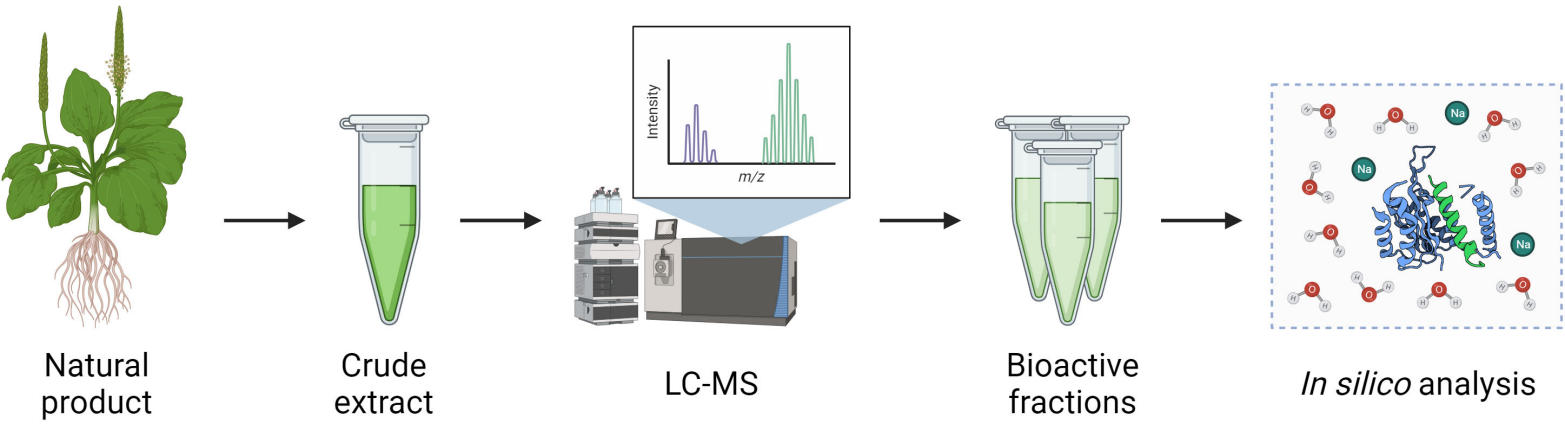
Pipeline for isolation of bioactive compounds from natural products. Crude extracts of NPs are obtained by a variety of methods such as ultrasonic or homogeniser-assisted extraction and are then separated into fractions and individual compounds using techniques such as LC-MS. Isolated compounds may then be further analysed using *in silico* modelling techniques.

In general, the discovery of bioactive molecules from NPs follows a pipeline involving the bioactivity-guided fractionation of crude NP extracts (**Figure 3**) (Atanasov et al., 2021). Firstly, the product is processed to isolate crude extracts containing complex mixtures of organic compounds with varying biochemical properties. The composition of these extracts can vary depending on the inherent hydrophilicity or lipophilicity of the solvent used and the method of extraction itself. This was demonstrated by El Maaiden et al., who compared a range of traditional and modern extraction methods to isolate bioactive compounds from dried plant material, and found that while modern techniques including microwave, ultrasonic, and homogenizer-assisted extraction gave consistently greater yields, compounds isolated using traditional techniques such as percolation and decoction had greater bioactivity, suggesting these methods may have induced fewer changes to the biochemical properties of the compounds (El Maaiden et al., 2022). Following extraction, *in vitro* assays are used to identify extracts with desirable bioactivity which are then separated into fractions using chromatographical techniques such as liquid chromatography-mass spectrometry (LC-MS). This process is repeated until bioactive compounds are isolated, which may then be further studied using *in silico* techniques such as molecular dynamics or molecular docking simulations to define their interactions with the environment and other molecules (Atanasov et al., 2021).

When considering viral entry inhibitors, one particularly useful assay for *in vitro* screening of drug candidates is the pseudovirus assay. This assay involves the creation of virus-like ‘pseudovirus’ particles comprising the core of a replication-deficient viral vector, often a lentivirus such as HIV-1, with incorporated surface glycoproteins of the virus of interest. Rather than delivering a viral genome, these pseudovirus particles deliver a reporter gene such as green fluorescent protein (GFP) or luciferase to a cell line expressing the viral receptor. Reduced expression of this reporter gene in the presence of potential entry inhibitors implies successful inhibition of entry. Further analysis including LC-MS and molecular docking may then clarify the bioactive molecules responsible for this bioactivity and characterize their interaction with the viral surface glycoprotein or cellular receptor. Pseudovirus assays are particularly useful when studying highly transmissible viruses such as SARS-CoV-2, as they allow relatively accurate simulation of the process of viral entry and reduce the need to use a replicating virus (Nie et al., 2020). These assays are routinely used to evaluate the neutralizing capability of patient sera against viruses including SARS-CoV-2, Ebola, Marburg, and Chikungunya virus (Liu et al., 2017; Wu et al., 2017; Zhang et al., 2017; Nie et al., 2020; Donofrio et al., 2021). Furthermore, many recent studies have utilized pseudovirus assays in their analyses of potential NP-derived inhibitors of SARS-CoV-2 viral entry, particularly those derived from plants (**Table 2**) (Goc et al., 2021b; Li et al., 2021; Mei et al., 2021; Zhan et al., 2021; Zhang et al., 2021; González-Maldonado et al., 2022; Wang et al., 2022; Yi et al., 2022; Meng et al., 2023; Risener et al., 2023). While other classes of NPs such as honey and marine sponge metabolites show promising anti-entry activity, further research including analysis using methods such as pseudovirus assays is required to validate their efficacy, particularly against new variants of SARS-CoV-2.

**Table 2:** Studies screening plant-derived NPs for SARS-CoV-2 S-ACE2 inhibitory activity.

Authors	Study design	Natural product	Analytical techniques	Summary of major findings
Al-Shuhaib et al. (2022)	*In silico*	Iraqi medicinal plants	Molecular docking, molecular dynamics	Epicatechin from *Hypericum perforatum* displayed high affinity for ACE2, interacting strongly and stably with residues directly involved in interaction with S RBD.
Campos et al. (2023)	*In vitro*	Bark extracts of *Ampelozizyphus amazonicus*	LC-MS, antibody-based inhibitor screening kit, infectivity assay	Aqueous and ethanol extracts inhibited the formation of the S-ACE2 complex by at least 50% and inhibited SARS-CoV-2 replication *in vitro*. The most promising EC_50_ values of extracts were <25 µg/ml. Phytochemical analysis identified a range of bioactive compounds including saponins, triterpenes, and phenolic compounds.
Chen C. et al. (2022)	*In silico*	Bioactive cannabinoids	Molecular docking, molecular dynamics	Luteolin, cannabigerovarinic acid (CBVGA), and cannabinolic acid (CBNA) had the highest affinity for the S-ACE2 complex out of the 42 cannabinoids screened. Based on interactions with key residues of the S RBD, luteolin and CBNA may inhibit SARS-CoV-2 entry.
Chen GY. et al. (2022)	*In vitro* and *in silico*	Database of extracts and compounds	Isothermal titration calorimetry (ITC), molecular docking, molecular dynamics, pseudovirus assay	39 compounds with affinity for S RBD were identified. Of these compounds, 10 µM dioscin, celastrol, epimedin C, amentoflavone, torvoside K, and saikosaponin C consistently inhibited 50-90% of spike pseudovirus entry into 293T-ACE2 cells.
El Hawary et al. (2020)	*In silico*	Phytocompounds from *Tecoma* spp.	LC-MS, molecular docking	12 phytocompounds isolated from eight *Tecoma* species showed moderate to strong binding affinity with the S-ACE2 interface. Succinic acid displayed the strongest affinity (-6.77 kcal/mol).
Gao et al. (2023)	*In vitro*	Japanese honeysuckle (*Lonicera japonica*)	LC-MS, antibody-based inhibitor screening kit, ACE2 activity assay,	Water and ethanol extracts inhibited the S-ACE2 interaction (65%, 100%) and ACE2 activity (90%, 62%). 36 bioactive compounds identified in extracts including flavonoids quercetin and luteolin as well as 10 novel compounds.
Goc et al. (2021b)	*In vitro*	Polyphenols and plant extracts	Antibody-based inhibitor screening kit, pseudovirus assay, cell fusion assay, TMPRSS2, CatL, and ACE2 activity assays, ACE2 binding assay, endosomal/lysosomal pH assay	Brazilin, theaflavin-3,3′-digallate (TF-3), and curcumin had strong affinity for S RBD, blocked pseudovirus fusion and entry, and reduced TMPRSS2 activity. TF-3 and curcumin also decreased ACE2 and CatL activity. TF-3 also decreased CatL expression by increasing endosomal pH.
González-Maldonado et al. (2022)	*In vitro*	Plant and fungal extracts, essential oils	Pseudovirus assay	*Stachytarpheta cayennensis* extract reduced D614G spike pseudovirus infectivity with an IC_50_ of 91.65 µg/ml. β-caryophyllene and *Phoradendron liga* extract also significantly decreased pseudovirus infectivity. Essential oils tested displayed no inhibitory effect and some appeared to increase infectivity.
Invernizzi et al. (2022)	*In vitro*	*Gunnera perpensa*	Bead-based protein binding assay (AlphaScreen), LC-MS	*G. perpensa* extract inhibited the S-ACE2 interaction with an IC_50_ of <0.001 µg/ml. Chromatographical techniques identified the ellagitannins punicalin and punicalagin as major constituents of *G. perpensa*. These isolated compounds inhibited the S-ACE2 interaction with respective IC_50_ values of 9 nM and 29 nM.
Kim et al. (2021)	*In vitro* and *in silico*	Geraniin	Antibody-based inhibitor screening kit, biolayer interferometry (BLI), molecular docking, molecular dynamics	Geraniin inhibited the S-ACE2 interaction (IC_50_ = 4.2 µM) and displayed strong affinity for both S RBD and ACE2 through interactions with multiple residues, although affinity for S RBD was higher.
Kumar et al. (2023)	*In silico*	Flavonoids	Molecular docking, molecular dynamics	Of the 40 flavonoids tested against the Omicron S RBD, five (tomentin A, tomentin C, hyperoside, catechin gallate, and corylifol A) displayed favorable properties including strong binding affinity, stable interaction dynamics, and favorable free binding energies.
Li et al. (2021)	*In vitro* and *in silico*	Glycyrrhizic acid	Pseudovirus assay, biotin binding assay, surface plasmon resonance (SPR) analysis, molecular docking	Glycyrrhizic acid (GA) inhibited the S-ACE2 interaction and pseudovirus entry. Pre-treating pseudovirus with GA had a greater effect than pre-treating cells, suggesting preferential binding with S rather than ACE2. Multiple GA binding pockets were identified on S RBD.
Mei et al. (2021)	*In vitro* and *in silico*	Chinese ephedra (*Ephedra sinica*)	Biotin binding assay, SPR analysis, LC-MS, molecular docking, pseudovirus assay	*Ephedra sinica* extract (ESE) inhibited the S-ACE2 interaction with an IC_50_ of 95.01 µg/ml and promoted virus-host dissociation. Quinoline-2-carboxylic acids were identified as major chemical constituents of ESE and could block S-ACE2 independently with micromolar IC_50_ values by interacting with residues in S RBD. These compounds also inhibited pseudovirus entry into multiple cell lines.
Meng et al. (2023)	*In vitro* and *in silico*	Flavonoids	Pseudovirus assay, BLI, molecular docking	24 of 31 tested flavonoids inhibited pseudovirus entry by binding to S RBD and blocking the S-ACE2 interaction, with myricetin having the most potent activity (IC_50_ = 10.27 ± 2.32 µM) and the highest affinity (K_D_ = 9.62 ± 2.11 µM). Molecular docking determined that flavonoids could interact with a highly conserved pocket in the S RBD, highlighting their potential for pan-variant entry inhibition.
Mhatre et al. (2021)	*In silico*	Catechins	Molecular docking, molecular dynamics	All tested catechins bound strongly to the S protein of wild-type and Alpha E484K variants. Epigallocatechin gallate (EGCG) had the strongest affinity for and the most stable interaction with both variants.
Mondal et al. (2021)	*In silico*	Biflavone-based antioxidants	Molecular docking	The natural anti-HIV agents hinokiflavone and robustaflavone displayed strong binding affinity for the S2 subunit of the S protein, interacting strongly with the HR1 and HR2 regions, suggesting potential for the inhibition of viral membrane fusion.
Nag et al. (2022)	*In silico*	Curcumin and other phytochemicals	Molecular docking, molecular dynamics	Curcumin interacted strongly and stably with multiple mutated residues in Omicron S RBD and S-ACE2 complex. The interaction also induced structural changes in S, possibly contributing to reduced affinity for ACE2.
Ohishi et al. (2022)	*In vitro* and *in silico*	Phytochemicals	ELISA, molecular docking, infectivity assay	Of the 10 phytochemicals tested, EGCG had the highest inhibitory activity against the S-ACE2 interaction (93.3%, 100 µM, IC_50_ = 33.9 µM). Curcumin also showed moderate activity (67%, 100 µM). EGCG interacted strongly with residues at the S-ACE2 interface and prevented SARS-CoV-2 infection *in vitro*.
Pan et al. (2023)	*In vitro* and *in silico*	Myricetin	Infectivity assay, molecular docking, BLI, pseudovirus assay, animal studies	Myricetin inhibited the replication of live SARS-CoV-2 (EC_50_ = 55.18 µM) and human coronavirus HCoV-229E (EC_50_ = 53.51 µM). The flavonoid also bound strongly to the S RBD of wild-type and mutated SARS-CoV-2, successfully blocking their interaction with ACE2. Pseudovirus entry into cells expressing ACE2 was also inhibited. Furthermore, myricetin exerted multiple anti-inflammatory effects in rat and mouse models.
Risener et al. (2023)	*In vitro*	Plant and fungal extracts	Pseudovirus assay, infectivity assay, LC-MS	*Solidago altissima*, *Salix nigra*, and *Pteridium aquilinum* extracts inhibited the entry of wild-type, Alpha, Beta, Gamma, and Delta spike pseudoviruses (EC_50_ <10 µg/ml). *S. altissima* and *P. aquilinum* extracts blocked the entry of replicating virus into Vero cells. Phenylpropanoids, flavonoids, and triterpenes were identified as the major phytochemical constituents in *S. altissima* and *P. aquilinum*.
Shin-Ya et al. (2023)	*In vitro* and *in silico*	Green tea, Matcha, black tea	Infectivity assay, antibody-based inhibitor screening kit, molecular docking	All three tea varieties effectively decreased the infectivity of Omicron subvariants BA.1, BA.2, XE, BA.5, BA.2.75, XBB.1, and BQ.1.1. Bioactive components of tea including the catechin EGCG and the theaflavin TFDG also decreased variant infectivity and blocked the interaction of BA.1 S RBD with ACE2.
Solo and Doss (2021)	*In silico*	North-East Indian medicinal plants	Molecular docking, molecular dynamics	50 phytochemicals from various plants displayed high binding affinity for Delta variant S. Of these, the top 15 compounds were flavones. 3,5,3′-Trimethoxy-6,7:4′,5′-bis(methylenedioxy)flavone from *Nicotiana plumbaginifolia* had the highest overall affinity for the S RBD (-8.7 kcal/mol).
Suručić et al. (2022)	*In vitro* and *in silico*	*Alchemilla viridiflora*	LC-MS, molecular docking, molecular dynamics, infectivity assay	Ellagitannins and flavonoids isolated from *A. viridiflora* extracts interacted favorably with S protein residues, with quercetin-3-(6”-ferulylglucoside) displaying the highest affinity (-8.035 kcal/mol). *A. viridiflora* methanol extract was also able to block SARS-CoV-2 entry *in vitro* (87.1%).
Wang et al. (2022)	*In vitro* and *in silico*	Peimine and other phytochemicals	Pseudovirus assay, fluorescence resonance energy transfer (FRET) assay, LC-MS, molecular docking	Peimine inhibited the entry of spike pseudoviruses into multiple cell lines by disrupting protein-protein interactions at the S-ACE2 interface. Activity extended to wild-type, B.1.1.7, and 501Y.V2 variants with EC_50_ values of 0.45 µM, 0.42 µM, and 0.43 µM respectively. Crude *Fritillaria* spp. extracts containing varying concentrations of peimine also inhibited entry.
Yi et al. (2022)	*In vitro* and *in silico*	Triterpenoids from Chinese liquorice (*Glycyrrhiza uralensis*)	Molecular docking, ELISA, pseudovirus assay, infectivity assay, immunofluorescence assay (IFA), SPR analysis	Glycyrrhetinic acid (GA) and licorice-saponin A3 (A3) displayed strong affinity for S RBD and inhibited the S-ACE2 interaction with respective IC_50_ values of 10.9 µM and 8.3 µM. GA and A3 also inhibited pseudovirus entry (EC_50_ = 4.98 µM, 9.30 µM) and live SARS-CoV-2 entry (EC_50_ = 3.17 µM, 75 nM).
Zhan et al. (2021)	*In vitro* and *in silico*	Flavonoids from sea buckthorn	Chromatography, SPR analysis, pseudovirus assay, molecular docking	Quercetin and isorhamnetin, major active flavonoids in sea buckthorn, displayed high affinity for ACE2. Isorhamnetin had the stronger affinity (K_D_ = 2.51 ± 0.68 µM) and interacted with three key residues of ACE2, enabling it to block the entry of spike pseudovirus.
Zhang et al. (2021)	*In vitro* and *in silico*	Library of NP-derived small molecules	Molecular docking, BLI assay, antibody-based inhibitor screening kit, pseudovirus assay, infectivity assay	14 compounds interacted strongly with both S RBD and ACE2. Of these, eight compounds blocked the S-RBD interaction. EGCG, isobavachalcone (Ibvc) and salvianolic acid A (SalA) blocked infection of HEK293 cells by D614G, N501Y, N439K, and Y453F variant spike pseudoviruses and live SARS-CoV-2 by targeting three pockets on the S RBD and interacting with ACE2. The most potent inhibitor, EGCG, inhibited pseudovirus entry with an EC_50_ of 0.3209 µM and live SARS-CoV-2 entry with an EC_50_ of 9.415 µM.
Zhu et al. (2023)	*In vitro* and *in silico*	Luteolin	ELISA, pseudovirus assay, SPR analysis, ACE2 activity assay, molecular docking	Luteolin significantly inhibited the S-ACE2 interaction (IC_50_ = 0.61 mM) and reduced Delta and Omicron pseudovirus entry by disrupting interactions between key residues. The flavonoid bound to both S RBD and ACE2, with a slight preference for ACE2. Luteolin also significantly reduced the activity of ACE2.

### 2.1 Coronavirus entry inhibitors from plants

Plants produce a wealth of bioactive compounds, many of which have been used in traditional medicines for thousands of years. By common definition, compounds produced by plants, referred to as phytochemicals, can be categorized either as primary metabolites, which are essential for growth, secondary metabolites, which mediate interactions with the environment including plant defense, or hormones, which regulate metabolism, although in reality there is much overlap between these groups (Erb and Kliebenstein, 2020). The therapeutic benefits of plant-derived NPs are often attributed to secondary metabolites such as polyphenols, alkaloids, and terpenoids. Polyphenols are the largest group of secondary metabolites and include aromatic compounds such as flavonoids, catechins, and tannins, while terpenoids and the nitrogen-containing alkaloids include compounds such as sesquiterpenes and quinine respectively (Cheng et al., 2007; Matsuura and Fett-Neto, 2017; Singh et al., 2021). These compounds have broad functionality in nature, including contributing to the color and aroma of plants, attracting pollinators, and defending against both biotic and abiotic stress (Cheng et al., 2007; Matsuura and Fett-Neto, 2017; Singh et al., 2021). Due to the extensive use of plant-derived NPs in traditional medicines and their contribution to many Western pharmaceuticals, there has been a substantial effort to characterize the anti-coronavirus properties of plant-derived NPs, including several studies evaluating their S-ACE2 inhibitory activity (**Table 2**). Gao et al. screened water and ethanol extracts of honeysuckle (*Lonicera japonicae*) using a S-ACE2 inhibitor screening kit, finding that both extracts inhibited the interaction significantly (65%, 100%) (Gao et al., 2023). Analysis of the chemical composition of these extracts revealed a variety of bioactive compounds including quercetin, luteolin, and 10 novel compounds (Gao et al., 2023). Studies such as these that focus on determining the bioactivity and composition of crude extracts are an important preliminary step in the pipeline of drug discovery from NPs, although the chemical and functional complexity of crude extracts limits the usefulness of such results without deeper analysis of the bioactivity of isolated compounds. For example, Yi et al. used a combination of *in vitro* and *in silico* methods to screen 125 compounds from *Glycyrrhiza uralensis*, a flowering plant used in traditional Chinese medicine, for their ability to block SARS-CoV-2 viral entry (Yi et al., 2022). Molecular docking analysis identified a number of triterpenoid saponins with strong affinity for the S RBD, and subsequent analyses determined that licorice-saponin A3 (A3) and glycyrrhetinic acid (GA) were capable of blocking SARS-CoV-2 pseudovirus entry by targeting the S RBD with potent IC_50_ values of 9.30 μM and 4.30 μM respectively (Yi et al., 2022). Interestingly, A3 had a much more potent inhibitory activity of 75 nM when screened using a SARS-CoV-2 infectivity assay, suggesting that the compound may have multiple viral targets. Subsequent analysis determined that A3 could bind very strongly with the nsp7 subunit of RdRp with a *K_D_* value of 167 nM, suggesting that this non-structural protein may be an additional viral target of A3 (Yi et al., 2022).

Similarly, Meng et al. used a variety of methods including a lentivirus-based pseudovirus assay to screen 31 flavonoids for their ability to block the S-ACE2 interaction. While most compounds exhibited some level of antiviral activity, myricetin was the most potent, blocking SARS-CoV-2 pseudovirus entry into a HEK293T cell line expressing ACE2 with an IC_50_ of 10.27 ± 2.32 µM (Meng et al., 2023). Furthermore, molecular docking analysis of flavonoids against the S proteins of wild-type, Delta, Omicron BA.1 and BA.2 SARS-CoV-2 variants identified a conserved binding pocket within the S protein RBD capable of forming stable interactions with myricetin, quercetin, and kaempferol, highlighting the potential of these compounds in the development of pan-variant S-ACE2 inhibitors (Meng et al., 2023). These findings were supported by Pan et al., who demonstrated the ability of myricetin to block the entry of SARS-CoV-2 (EC_50 _= 55.18 µM) as well as pseudoviruses expressing the wild-type, N501Y, N439K, Y453F, and D614G mutated spike proteins (Pan et al., 2023). This study also highlighted the anti-inflammatory properties of myricetin in mouse and rat models, demonstrating its strong potential as a COVID-19 therapeutic agent (Pan et al., 2023). Broadly speaking, flavonoids such as myricetin are some of the most promising natural drug candidates for antiviral development. Multiple studies have demonstrated the S-ACE2 inhibitory activity of compounds such as epicatechin, luteolin, curcumin, and quercetin using a variety of *in vitro* and *in silico* methodologies (Goc et al., 2021b; Zhan et al., 2021; Al-Shuhaib et al., 2022; Chen C. et al., 2022; Nag et al., 2022; Ohishi et al., 2022; Suručić et al., 2022; Gao et al., 2023; Zhu et al., 2023). Other polyphenolic compounds display similar potential, including ellagitannins and carboxylic acids which have some of the most potent S-ACE2 IC_50_ values in recent literature, often in the nanomolar range (**Table 3**) (Mei et al., 2021; Invernizzi et al., 2022). Invernizzi et al. screened the extract of the South African medicinal plant *Gunnera perpensa* against the S-ACE2 interaction, revealing potent inhibition (IC_50_ <0.001 µg/ml) (Invernizzi et al., 2022). The ellagitannins punicalin and punicalagin were identified as major chemical components and displayed respective IC_50_ values of 9 nM and 29 nM (Invernizzi et al., 2022). As such, further research is needed to identify the key components of crude extracts with strong bioactivity and to validate their drug potential through *in silico* and *in vivo* studies. Furthermore, it is essential that the influence of S RBD antigenic variation on the efficacy of such compounds be thoroughly investigated to gauge their potential for use against emerging variants of SARS-CoV-2 and other coronaviruses.

**Table 3:** NP-derived compounds with reported S-ACE2 inhibitory activity sorted by IC_50_.

Compound	Source	Reported IC_50_ (µM)	Reference
Punicalin	*Gunnera perpensa*	0.009	Invernizzi et al. (2022)
Punicalagin	*Gunnera perpensa*	0.029	Invernizzi et al. (2022)
4,6-dihydroxyquinoline-2-carboxylic acid	*Ephedra sinica*	0.07	Mei et al. (2021)
4-hydroxy-6-methoxyquinoline-2-carboxylic acid	*Ephedra sinica*	0.15	Mei et al. (2021)
4-hydroxyquinoline-2-carboxylic acid	*Ephedra sinica*	0.58	Mei et al. (2021)
Thorectidiol A	*Dactylospongia elegans*	1.0 ± 0.7	Williams et al. (2023)
Geraniin	*Elaeocarpus sylvestris* var. *ellipticus* and *Nepheliuim lappaceum*	4.2	Kim et al. (2021)
Licorice-saponin A3	*Glycyrrhiza uralensis*	8.3	Yi et al. (2022)
Myricetin	Common flavonoid	10.27 ± 2.32	Meng et al. (2023)
Glycyrrhetinic acid	*Glycyrrhiza uralensis*	10.9	Yi et al. (2022)
Quercetin	Common flavonoid	17.00 ± 3.42	Meng et al. (2023)
Epigallocatechin gallate	Common tannin	33.9	Ohishi et al. (2022)
Luteolin	Common flavonoid	610	Zhu et al. (2023)

### 2.2 Coronavirus entry inhibitors from honey

Like plants, honey has been used historically in traditional medicine due to its inherent antioxidative, anti-inflammatory, immunomodulatory and antimicrobial properties (Samarghandian et al., 2017). Certain varieties such as Manuka honey collected by monofloral honeybees in New Zealand have displayed *in vitro* virucidal activity against viruses such as influenza A, HIV-1, and varicella zoster virus (Watanabe et al., 2014; Shahzad and Cohrs, 2012; Obossou et al., 2022). While there has been relatively little research regarding the potential role of honey in countering SARS-CoV-2 infection, the phenolic content of certain varieties has been associated with the reduced expression of proinflammatory cytokines (Biluca et al., 2020; da Silva et al., 2022). Therefore, it is possible that components of honey may contribute to attenuation of the aggressive immune response seen in severe cases of COVID-19, although there are currently few published clinical trials investigating this. Ashraf et al. conducted a placebo-controlled randomized trial of the combined use of honey and *Nigella sativa* seeds in the treatment of COVID-19, finding that participants receiving the treatment experienced alleviation from symptoms approximately twice as fast as the placebo group, had a significantly shorter duration of viral load, and exhibited a lower mortality rate in severe cases (Ashraf et al., 2023). While promising, further research is required to verify these findings. Honey varieties contain a complex assortment of phytochemicals that vary based on many factors including the plant source and pollination method (Al-Hatamleh et al., 2020; Abedi et al., 2021). While this can complicate the isolation of compounds present in trace amounts, it is also conducive to diverse bioactivity including the inhibition of the S-ACE2 interaction. One study screened 12 honey samples produced by Indonesian stingless honeybees using a S-ACE2 inhibitor test kit and found that 10 samples produced an inhibitory effect of 50% or greater (Arung et al., 2022). Of these samples, the bitter variant of the honey produced by the honeybee *Wallacetrigona incisia* had the strongest activity, preventing 99.7% of S-ACE2 binding at 2% (v/v) in water (Arung et al., 2022). Interestingly, the pineapple-flavored variant of *W. incisia* honey had a much lower inhibitory activity of 50.3% at 2% concentration (Arung et al., 2022). Phytochemical analyses suggested that this difference may be due to the absence of coumarin, an aromatic organic compound commonly found in cinnamon (Arung et al., 2022). Thus, while honey represents a promising alternative source of bioactive compounds with antiviral properties, the diverse chemical composition between and within varieties complicates analysis. Ultimately, further research is required to elucidate the range of mechanistic effects honey may have on coronavirus infection and to better evaluate its potential contribution to the development of antivirals such as entry inhibitors.

### 2.3 Coronavirus entry inhibitors from marine sponges

Another source of natural compounds with antiviral properties is marine sponges. Compounds such as nucleosides, quinones, and alkaloids are found in marine sponges and have historically displayed antiviral activity, particularly against HIV-1 (Patil et al., 1992; Sagar et al., 2010; O'Rourke et al., 2016). Additionally, recent work has highlighted the potential use of such molecules in the development of SARS-CoV-2 antivirals. One study conducted *in silico* screening of the marine sponge metabolite ilimaquinone in comparison to repurposed antivirals including remdesivir, finding strong affinity for multiple viral proteins including S and PLpro (Surti et al., 2020). Another *in silico* study screened a broad library of marine alkaloids and found that the marine sponge metabolite 8-hydroxymanzamine displayed potential antiviral activity against 3CLpro (Swain et al., 2022). Marine sponge metabolites also show promise as S-ACE2 inhibitors. One study isolated and characterized thorectidiol A, a novel terpenoid with antiviral properties, from the marine sponge *Dactylospongia elegans*. Methanol extracts of *D. elegans* tissue were analyzed by chromatography to isolate thorectidiol A and its acetylated derivative thorectidiol A diacetate, which were then determined by means of a bead-based luminescence assay to have significant inhibitory activities against the S-ACE2 interaction with respective IC_50_ values of 1.0 ± 0.7 µM and 7.3 ± 2.6 µM (Williams et al., 2023). Subsequent larger-scale extraction of *D. elegans* tissue yielded the additional derivatives thorectidol acetate and thorectidiol B diacetate, which interestingly displayed negligible inhibitory activity. Thus, while marine sponge metabolites have established antiviral activity and show potential for use in the development of coronavirus entry inhibitors, there remains the need to characterize the structure-activity relationship of these compounds, and for more extensive *in vitro* and *in silico* analyses to validate their activity against SARS-CoV-2 variants.

## 3 Conclusion

While vaccination has effectively reduced the threat of COVID-19 in many parts of the world, there remain limited options for coronavirus-specific antivirals to protect those at risk of severe disease from current and emerging SARS-CoV-2 variants and to curb the severity of future coronavirus outbreaks. Of the potential mechanisms by which novel coronavirus antivirals may function, inhibiting entry by blocking the S-ACE2 interaction is a promising option supported by many *in vitro* and *in silico* studies. While such drugs may be developed by rational drug design, numerous recent studies have highlighted the *in vitro* efficacy of natural products derived from plants, honey, and marine sponge metabolites in blocking the S-ACE2 interaction and the cellular entry of both pseudoviruses and live SARS-CoV-2 variants. Given the advantages of NP-derived therapeutics, further research should be conducted to better understand the potential role of these compounds in the development of coronavirus S-ACE2 inhibitors, including the influence of pan-variant and pan-coronavirus antigenic variation on their efficacy, before progressing to *in vivo* studies. Furthermore, the antiviral activity of certain classes of NPs such as honey and marine sponge metabolites remains insufficiently researched in relation to coronavirus entry inhibition. In the future, such research may contribute to the development of antiviral therapies such as nasal sprays to be used as an additional precaution against infection or to prevent a cascade of disease severity in high-risk individuals. Such therapies have been trialed in recent years, including a nasal spray containing the algal metabolite iota carrageenan (Figueroa et al., 2021). This compound effectively inhibited SARS-CoV-2 cellular entry (IC_50_ = 4.947 µg/ml), and demonstrated *in vivo* efficacy in a clinical study aimed at protecting unvaccinated healthcare workers treating patients with COVID-19 (Figueroa et al., 2021; Varese et al., 2021). Thus, natural products show significant potential in antiviral development and should be thoroughly investigated given the urgent need to develop pan-coronavirus entry inhibitors in anticipation of future outbreaks of coronaviruses.

## Author contributions

DS: Writing – review & editing, Writing – original draft. AC: Writing – review & editing. CM: Writing – review & editing. PS: Conceptualization, Writing – review & editing.
