# Supplementary material for: Natural products as a source of Coronavirus entry inhibitors
Source: Front Cell Infect Microbiol. 2024 Feb 21;14:1353971. doi: 10.3389/fcimb.2024.1353971 (PMC10915212; doi:10.3389/fcimb.2024.1353971)
Supplement: Supplementary file 1 [file DataSheet_1.pdf]

Search methodology

Review question

To what extent do different classes of natural products show promise as sources of SARS-CoV-2 S-ACE2 fusion/entry inhibitors?

Searches

Databases
PubMed

Search terms
“SARS-CoV-2” OR “COVID-19” OR “coronavirus” AND “entry inhibitor” OR “fusion inhibitor” OR “S-ACE2” OR “spike” OR “ACE2” AND “natural product” OR “plant” OR “botanical” OR “honey” OR “marine sponge” OR “screening”

Results limited to English language only
No date limitations applied

Inclusion/exclusion criteria

Inclusion
In vitro studies screening broad libraries of NPs, *in vitro* studies of specific NPs, *in vitro* or *in silico* studies of NP-derived compounds, randomised controlled trials. This review is limited to studies focusing on NPs or NP-derived compounds with reported SARS-CoV-2 entry inhibition activity via disruption of the S-ACE2 interaction. Major focus areas were plants, honey, and marine sponge metabolites.

Exclusion
Preprints and other grey literature.
